# Clinical Characteristics of Children With COVID-19 in the United Arab Emirates: Cross-sectional Multicenter Study

**DOI:** 10.2196/29049

**Published:** 2021-11-05

**Authors:** Farah Ennab, Mariam ElSaban, Eman Khalaf, Hanieh Tabatabaei, Amar Hassan Khamis, Bindu Radha Devi, Kashif Hanif, Hiba Elhassan, Ketharanathan Saravanan, David Cremonesini, Rizwana Popatia, Zainab Malik, Samuel B Ho, Rania Abusamra

**Affiliations:** 1 College of Medicine Mohammed Bin Rashid University of Medicine and Health Sciences Dubai United Arab Emirates; 2 Department of Pediatrics Mediclinic City Hospital Dubai United Arab Emirates; 3 Department of Pediatrics Mediclinic Welcare Hospital Dubai United Arab Emirates; 4 Department of Pediatrics Mediclinic Parkview Hospital Dubai United Arab Emirates; 5 Department of Medicine Mediclinic City Hospital Dubai United Arab Emirates

**Keywords:** pediatrics, children, COVID-19, SARS-CoV-2, United Arab Emirates, viral shedding, pandemic, treatment, outcomes, clinical, public heath

## Abstract

**Background:**

COVID-19 has infected over 123 million people globally. The first confirmed case in the United Arab Emirates (UAE) was reported on January 29, 2020. According to studies conducted in the early epicenters of the pandemic, COVID-19 has fared mildly in the pediatric population. To date, there is a lack of published data about COVID-19 infection among children in the Arabian region.

**Objective:**

This study aims to investigate the clinical characteristics, laboratory findings, treatment, and outcomes of children with COVID-19.

**Methods:**

This cross-sectional, multicenter study included children with confirmed COVID-19 infection admitted to 3 large hospitals in Dubai, UAE, between March 1 and June 15, 2020. Serial COVID-19 polymerase chain reaction (PCR) testing data were collected, and patients’ demographics, premorbid clinical characteristics, and inpatient hospital courses were examined.

**Results:**

In all, 111 children were included in our study and represented 22 nationalities. Of these, 59 (53.2%) were boys. The mean age of the participants was 7 (SD 5.3) years. About 15.3% of children were younger than 1 year. Only 4 (3.6%) of them had pre-existing asthma, all of whom had uneventful courses. At presentation, of the 111 children, 43 (38.7%) were asymptomatic, 68 (61.2%) had mild or moderate symptoms, and none (0%) had severe illness requiring intensive care. Fever (23/111, 20.7%), cough (22/111, 19.8%), and rhinorrhea (17/111, 15.3%) were the most common presenting symptoms, and most reported symptoms resolved by day 5 of hospitalization. Most patients had no abnormality on chest x-ray. The most common laboratory abnormalities on admission included variations in neutrophil count (22/111, 24.7%), aspartate transaminase (18/111, 22.5%), alkaline phosphatase (29/111, 36.7%), and lactate dehydrogenase (31/111, 42.5%). Children were infrequently prescribed targeted medications, with only 4 (3.6%) receiving antibiotics. None of the 52 patients tested for viral coinfections were positive. COVID-19 PCR testing turned negative at a median of 10 days (IQR: 6-14) after the first positive test. Overall, there was no significant difference of time to negative PCR results between symptomatic and asymptomatic children.

**Conclusions:**

This study of COVID-19 presentations and characteristics presents a first look into the burden of COVID-19 infection in the pediatric population in the UAE. We conclude that a large percentage of children experienced no symptoms and that severe COVID-19 disease is uncommon in the UAE. Various laboratory abnormalities were observed despite clinical stability. Ongoing surveillance, contact tracing, and public health measures will be important to contain future outbreaks.

## Introduction

The COVID-19 pandemic has created a global health care crisis, with over 123 million infections reported in more than 185 countries [1]. The death toll from the ongoing pandemic has crossed the 2-million mark and continues to rise [2]. Early studies reported a predominance of respiratory symptoms in adults and increased fatality among older individuals. As infection trends evolved, reports highlighted that other organ systems were also affected by COVID-19 infection. Pediatric COVID-19 studies from China [3], the United States [4], and Europe [5] have demonstrated similarities in disease prevalence, clinical characteristics, and outcomes. Although COVID-19 has fared mildly in the pediatric population, ongoing research is crucial to improve our understanding of this disease in various parts of the world and the role played by children in community-based viral transmission.

As is now widely known, the SARS-CoV-2 outbreak was first identified in December 2019 in Wuhan, China [6]. In contrast, the first confirmed case in the United Arab Emirates (UAE) was reported on January 29, 2020. Our study was conducted in the emirate of Dubai in the UAE, with a population of 3.35 million people from over 200 countries [7]. Dubai has a young population demographic, with 18% of its population aged 19 years or younger. In this study, we sought to determine whether pediatric COVID-19 infection in Dubai, with its unique population demographic, was similar to that reported in other parts of the world. UAE’s proactive public health approach, including early school closures from March 8, 2020, the suspension of public transport, mandatory mask-wearing in public, restrictions on family gatherings, 2-week sterilization campaigns, strict lockdown for containment of the virus, robust testing, and contact tracing played an important role in limiting the spread of COVID-19 infection in the UAE, especially among children. Our findings of COVID-19 infection among children in Dubai will provide a global perspective of disease trends caused by the novel coronavirus and help shape public health policies in the future.

## Methods

### Study Design and Recruitment

This cross-sectional, multicenter study was conducted across 3 large tertiary-care hospitals in Dubai, UAE. Our study population included a total of 111 consecutive pediatric patients admitted to the participating hospitals between March 1 and June 15, 2020. Children 18 years or younger with a confirmed diagnosis of COVID-19 were enrolled in the study. This study was reviewed and approved by the Mediclinic Middle East Institutional Review Board and the Dubai Health Authority’s Dubai Scientific Research Ethics Committee. The requirements for written consent were waived by the boards.

Infection was confirmed by qualitative detection of SARS-CoV-2 RNA using real-time reverse-transcription polymerase chain reaction (RT-PCR) through a simultaneous examination of ORF1ab and N-gene from nasopharyngeal swab samples. Patients were tested as a result of clinical symptoms suggestive of COVID-19 infection or a history of close contact with an individual with confirmed COVID-19 infection.

### Participants and Data Collection

Data on patient demographics and epidemiology; comorbidities; clinical characteristics; laboratory results, including COVID-19 PCR tests and radiographic findings; and hospital course, including treatment modalities and outcomes, were collected for all patients. BMI for age percentiles were based on calculators adapted from the Centers for Disease Control and Prevention (CDC) population standards for children and adolescents [8]. Classification of disease severity on admission was based on the most recent UAE National Guidelines for Clinical Management and Treatment of COVID-19 at the time [9]. Disease severity was classified into 4 types, as described below. First, *asymptomatic* cases were those with no clinical symptoms, normal inflammatory markers, and normal chest x-ray (CXR). Second, *mild* cases were those with any clinical symptoms (eg, sore throat, nasal congestion, cough, fatigue, myalgia, and fever), normal chest auscultation, normal inflammatory markers, and a normal CXR. Third, moderate cases were those including any of the following: CXR with infiltrates in <50% of lung fields, oxygen saturation (SpO_2_) <95% in room air, mild to moderate tachypnea, or elevated inflammatory markers (eg, lactate dehydrogenase [LDH] >245 IU/L, ferritin >300 ng/mL, lymphopenia <0.8 × 10^9^/L , c-reactive protein [CRP] >100 mg/L. Fourth, severe cases were those with CXR with infiltrates in >50% of lung fields, SpO_2_ <92% in room air or requiring >4 L/min of supplemental oxygen to maintain SpO_2_ >94%, tachypnea, respiratory alkalosis, respiratory acidosis, metabolic acidosis, the ratio of arterial oxygen partial pressure (PaO_2_ in mmHg) to fractional inspired oxygen (PaO_2_/FiO_2_) <300 or SpO_2_/FiO_2_ ratio <264, or any of the following complications: severe pneumonia, acute respiratory failure and acute respiratory distress syndrome , acute renal failure, disseminated intravascular coagulation, sepsis or septic shock.

### Statistical Analysis

Data were collected from the patients’ electronic medical records and paper charts, entered into Microsoft Excel, and independently reviewed by 4 coinvestigators to verify data accuracy. Data were analyzed using the Statistical Package for Social Sciences (SPSS) software (version 25.0; IBM Corp). Frequencies with proportions were reported for categorical variables, and means with SDs were reported for continuous variables. Association between categorical variables was tested by the chi-square and Fischer Exact test when appropriate. Mann-Whitney *U* test was used to compare means between 2 groups, and the Kruskal-Wallis H test was used to compare means between more than 2 groups. A *P* value <.05 was considered statistically significant.

## Results

### Overview

A total of 111 children, aged 18 years or below, were hospitalized with COVID-19 at one of the participating hospitals during the study period between March 1 and June 15, 2020. Over the same period, 1422 adults with COVID-19 were admitted at the 3 study hospitals. Children constituted 7.8% of the total COVID-19 hospital admissions during the study period. Their mean age was 7 years (range: 17 days to 17.2 years). Our analysis showed that significantly more children aged 6 years or below had COVID-19–related symptoms compared to older children (who were more likely to be asymptomatic). Boys were slightly overrepresented in our sample, with a boy:girl ratio of 1.13. Information regarding BMI was available for only 42 of the 111 (37.8%) children, and about half of them (22/42, 52.3%) had BMI measurements within the normal range for age. Underlying chronic health conditions were infrequently reported. Our patient population comprised a total of 22 different nationalities, with the top 5 nationalities being India (35/111, 31.5%), UAE nationals (27/111, 24.3%), Filipinos (15/111, 13.5%), Egyptians (6/111, 5.4%) and Pakistanis (5/111, 4.5%). The vast majority of our patients had a history of household or family exposure to an adult with confirmed COVID-19 diagnosis, and travel outside the UAE in the preceding 2 weeks was an infrequent risk factor for exposure (Table 1).

**Table 1:** Demographic and epidemiological characteristics of children with COVID-19.

Characteristics		Total participants, n (%) (N=111)	Asymptomatic, n (%) (n=43)	Symptomatic, n (%) (n=68)	*P* value	
**Age (years) **						.02
	≤1	17 (15.3)	6 (14)	11 (16.2)		
	1-6	36 (32.4)	8 (18.6)	28 (41.2)		
	6-12	32 (28.8)	19 (44.2)	13 (19.1)		
	≥12	26 (23.4)	10 (23.3)	16 (23.5)		
**Gender **						.15
	Boy	59 (53.2)	26	33		
	Girl	52 (46.8)	17	35		
**BMI^a^**						.37
	Underweight	7 (16.7)	1 (6.3)	6 (23.1)		
	Normal	22 (52.3)	8 (50)	14 (53.8)		
	Overweight	7 (16.7)	4 (25)	3 (11.5)		
	Obese	6 (14.3)	3 (18.8)	3 (11.5)		
**Nationality **						.18
	Emirati	27 (24.3)	13 (30.2)	14 (20.6)		
	Expatriates	84 (75.7)	30 (69.8)	54 (79.4)		
**Pre-existing medical conditions **						
	Asthma	4 (3.6)	1(2.3)	3 (4.4)	.50	
	Prematurity^b^	2 (1.8)	1 (2.3)	1 (1.5)	.63	
	Diabetes mellitus (type 1)	2 (1.8)	2 (4.7)	0	.15	
**Epidemiological history **						
	Close contact^c^	104 (93.7)	41 (95.3)	63 (92.6)	.44	
	Travel outside the UAE	4 (3.6)	0	4 (5.9)	.14	

^a^BMI was calculated for 42 children ≥2 years for whom height and weight data were available. It was defined as percentiles for age as per the Centers for Disease Control and Prevention guidelines for children, as follows: underweight <5th percentile; normal ≥5th to <85th percentile; overweight ≥85th to <95th percentile; and obese ≥95th percentile.

^b^Prematurity per the World Health Organization subcategory of very preterm babies (28-32 weeks).

^c^Close contact was defined as being in contact with someone with confirmed COVID-19 for over 15 minutes.

### Spectrum of Clinical Symptoms

**Figure 1:**
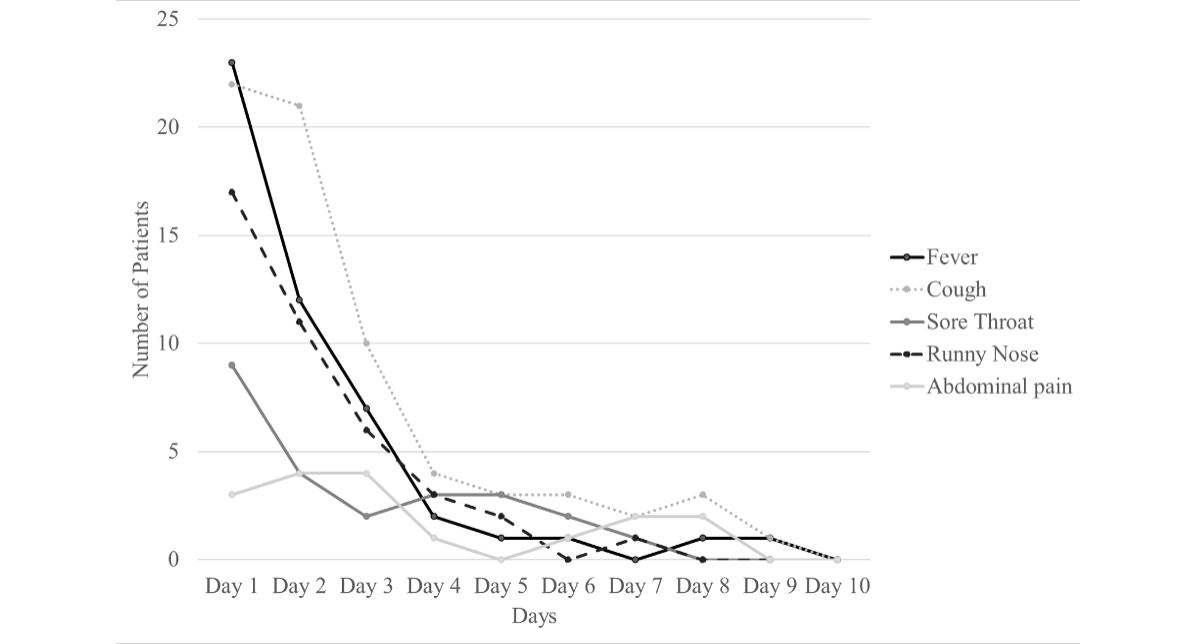
Trends in clinical symptoms during hospitalization.

A total of 61.2% (68/111) children presented with mild or moderate symptoms. There were no children admitted with severe symptoms during our study. Fever, cough, and rhinorrhea were the most common presenting symptoms among our patients (Table 2). Anosmia, rash, and gastrointestinal symptoms were infrequently reported on admission. Most of these symptoms had resolved by day 5 of hospitalization (Figure 1). None of the children presented with signs or symptoms suggestive of neurological, cardiac, or renal dysfunction.

**Table 2:** Clinical symptoms and severity classification on admission.

		Participants, n (%)
**Clinical symptoms**		
	Fever	23 (20.7)
	Cough	22 (19.8)
	Rhinorrhea	17 (15.3)
	Myalgia or fatigue	9 (8.1)
	Sore throat	9 (8.1)
	Headache	6 (5.4)
	Anosmia	5 (4.5)
	Abdominal Pain	3 (2.7)
	Nausea or vomiting	3 (2.7)
	Diarrhea	2 (1.8)
	Rash	1 (0.9)
	Dyspnea	0 (0)
**Classification of clinical severity^a^**		
	Asymptomatic	43 (38.7)
	Mild	32 (28.8)
	Moderate	36 (32.4)
	Severe	0 (0)

^a^Classification was based on the United Arab Emirates National Guidelines for Clinical Management and Treatment of COVID-19, April 2020.

### Radiologic and Laboratory Findings

Overall, 94 (84.7%) children had chest imaging performed during their hospitalization; the vast majority of which was CXR. Only 2 (1.8%) children had chest computerized tomography (CT) scans, of which 1 child had both CXR and chest CT scans performed. In all, 12 (10.8%) children had 2 CXRs performed over the course of hospitalization. Prominent bronchovascular markings were the most frequently reported CXR findings. Interstitial infiltrates were noted for 7 children (7.5%), 4 of whom had bilateral infiltrates; 4 (3.6%) had bronchial thickening, and only 1 (0.9%) child had ground-glass appearance on CXR. Consolidation or nodular changes on CXR were not reported for any children.

Elevated aspartate transaminases (AST), alkaline phosphatase (ALP), and LDH levels were the most encountered abnormal tests on admission (Table 3). Subgroup analysis of laboratory findings showed that symptomatic patients had significantly higher CRP and LDH and lower hemoglobin when compared to asymptomatic patients.

**Table 3:** Laboratory parameters on hospital admission.

Laboratory parameter	Value, median (range)				*P* value		Abnormal results^a^, n (%)
	Total (N=111)	Asymptomatic (n=43)	Symptomatic (n=68)				
Total WBC^b^ in ×10^9^/L (n=89)	7.0 (3.0-17.5)	6.6 (3.9-13.1)	7.2 (3.0-17.5)	.85		11 (12.4)	
Neutrophils in ×10^9^/L (n=89)	2.3 (0.2-8.11)	2.5 (0.2-8.1)	2.2 (0.3-6.1)	.22		22 (24.7)	
Lymphocytes in ×10^9^/L (n=89)	3.43 (1.1-12.8)	3.0 (2.03-11)	3.7 (1.1L (n=89)-12.8)	.22		7 (7.9)	
Hemoglobin in g/dL (n=89)	12.8 (9.5-18.8)	13 (11-18.8)	12.3 (9.5-16.7)	.006		9 (10.1)	
Platelets in ×10^9^/L (n=89)	283 (133-562)	280 (133-510)	283.5 (182-562)	.89		8 (9.9)	
Sodium in mmol/L (n=81)	140 (130-144)	140 (131-144)	140 (130-144)	.28		2 (2.5)	
Potassium in mmol/L (n=81)	4.3 (3.4-5.8)	4.1 (3.4-5.8)	4.4 (3.5-5.3)	.16		6 (7.5)	
Calcium in mmol/L (n=46)	2.42 (2.22-2.73)	2.42 (2.23-2.73)	2.42 (2.22-2.67)	.96		0 (0)	
Magnesium in mmol/L (n=27)	0.86 (0.79-1.01)	0.86 (0.79-0.98)	0.89 (0.81-1.01)	.37		0 (0)	
Creatinine in mmol/L (n=23)	51 (35-84)	54 (35-84)	50 (39-73)	.98		5 (21.7)	
AST^c^ in IU/L (n=80)	25 (12-114)	24 (14-59)	26 (12-114)	.10		18 (22.5)	
ALT^d^ in IU/L (n=80)	15 (8-76)	15.5 (9-49)	15 (8-76)	.51		1 (1.3)	
ALP IU/L (n=79)	211 (37-430)	191 (55-372)	218 (37-430)	.29		29 (36.7)	
Albumin in g/dL (n=80)	43 (35.3-49.1)	43.6 (36.5-48)	42.5 (35.3-49.1)	.10		3 (3.7)	
Amylase in IU/L (n=35)	66 (23-136)	65 (23-136)	69.5 (31-131)	.33		3 (8.6)	
Lipase in IU/L (n=34)	18 (7-64)	17 (13-45)	19.5 (7-64)	.96		1 (2.9)	
INR^e^ (n=52)	1.04 (0.10-1.34)	1.04 (0.88-1.34)	1.02 (0.10-1.3)	.72		3 (5.8)	
PT^f^ in seconds (n=52)	13.1 (10.9-15.7)	13.1 (10.9-15.7)	13.1 (11-15.3)	.69		1 (1.9)	
aPTT^g^ in seconds (n=54)	34.25 (26.9-60.6)	33.4 (27.7-43.2)	34.6 (26.9-60.6)	.25		3 (5.8)	
Fibrinogen in mg/dL (n=34)	299 (228-986)	282.5 (228-465)	305.5 (246-986)	.14		2 (3.7)	
CRP^h^ in mg/dL (n=85)	1.0 (0.10-183.6)	0.9 (0.10-19.5)	1.0 (0.10-183.6)	.047		11 (12.9)	
LDH^i^ in IU/L (n=73)	232 (134-493)	204.5 (134-245)	258 (142-493)	<.001		31 (42.5)	
Procalcitonin in ng/mL (n=61)	0.05 (0.02-0.45)	0.05 (0.02-0.07)	0.05 (0.02-0.45)	.28		0 (0)	
D-dimer in ng/mL (n=55)	270 (18-3232)	229.5 (56-3232)	300 (10-1140)	.22		10 (18.2)	
Ferritin in ng/mL (n=74)	39.6 (6.66-1276.6)	39.7 (17.7-97.7)	39.4 (6.7-127.8)	.89		5 (6.8)	
Creatine kinase IU/L (n=41)	96 (4.3-221)	99.5 (42-163)	76 (4.3-221)	.38		2 (4.9)	

^a^Abnormal values based on our laboratory age-specific ranges.

^b^WBC: white blood cells.

^c^AST: aspartate transaminase.

^d^ALT: alanine transaminase.

^e^INR: international normalized ratio.

^f^PT: prothrombin time.

^g^aPTT: activated partial thromboplastin time.

^h^CRP: C-reactive protein.

^i^LDH: lactate dehydrogenase.

### Treatment, Clinical Course, and Virologic Outcomes

Children received treatment for COVID-19 according to the UAE National Guidelines published at the time [9]. Hydroxychloroquine was given for a mean of 4.9 days and azithromycin for a mean of 4.8 days. Overall, these medications were well tolerated, and only 1 (5.8%) child reported adverse reactions to hydroxychloroquine (nausea and vomiting) and 1 (25%) to azithromycin (vomiting). One child received both lopinavir-ritonavir and systemic corticosteroids. Patients in our study were infrequently treated for bacterial coinfections, and there was no significant difference in treatment between symptomatic and asymptomatic groups (Table 4).

**Table 4:** Treatments and complications during hospital stay.

		Total cohort (N=111)	Asymptomatic (n=43)	Symptomatic (n=68)	*P* value
**Treatment, n (%) **					
	Hydroxychloroquine	17 (15.3)	5 (11.6)	12 (17.6)	.28
	Azithromycin	4 (3.6)	2 (4.7)	2 (2.9)	.50
	Antibiotics	6 (5.4)	3 (7)	3 (4.4)	.16
	Lopinavir-ritonavir	1 (0.9)	0 (0)	1 (1.5)	.61
	Steroids	1 (0.9)	0 (0)	1 (1.5)	.61
**Complications**					
	Pneumonia, n (%)	3 (2.7)	0 (0)	3 (100)	N/A
	Duration of hospitalization days, median (range)	8 (0-30)	7 (1-25)	9 (0-30)	.19
**Outcome, n (%)**					
	Discharge	111 (100)	43	68	N/A^a^
	Deaths	0 (0)	0 (0)	0 (0)	N/A

^a^N/A: not applicable

**Figure 2:**
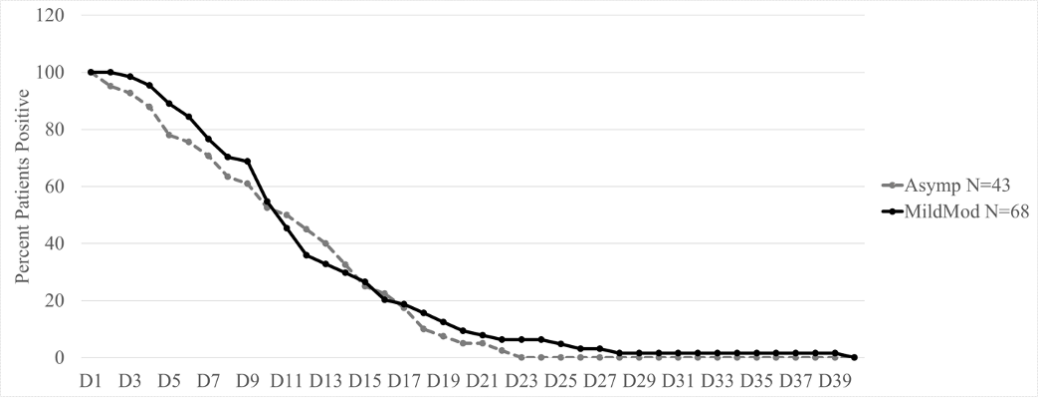
Positivity rate of polymerase chain reaction testing for COVID-19 during hospitalization. Asymp: asymptotic patients; MildMod: mild to moderate cases.

Children were discharged when clinically stable, and COVID-19 PCR test appeared negative as per the UAE National Guidelines for Clinical Management and Treatment of COVID-19 [9]. There were no deaths among our study patients. Among the 68 symptomatic patients in our study, 52 (76.4%) had their nasal samples sent for a respiratory viral PCR panel, and no viral coinfections were detected. Among our total study sample, COVID-19 PCR test results appeared negative after a median of 10 days (IQR 6-14) after the first positive test. There was no significant difference in the median duration of COVID-19 PCR positivity between symptomatic and asymptomatic patients (Figure 2).

D0 signifies the day of first positive COVID-19 PCR test. A positive PCR test result reverted to negative after a median of 10 days in both asymptomatic and symptomatic patients.

## Discussion

### Principal Findings

In this inaugural pediatric COVID-19 study from the UAE, we shared a comprehensive description of pediatric presentations of COVID-19 during the first wave in Dubai, UAE. This included providing a clear picture of the various ways in which children with COVID-19 can present, monitoring their clinical course, and assessing the total duration of the viral shedding period.

Our findings revealed that the majority of children in our sample size were either asymptomatic or had only mild to moderate symptoms. No cases of severe disease were reported in our sample. COVID-19 PCR turned negative at a median of 10 days after the first positive test. Overall, there was no significant difference in viral shedding duration between asymptomatic and symptomatic children.

A consideration to emphasize is the prevalence of COVID-19 testing in the UAE, which was among the highest reported globally [10], with comprehensive contact tracing that identifies a sizable number of asymptomatic individuals. The UAE National Guidelines followed during the study period required all COVID-19–positive individuals to be admitted to hospitals for the duration of their COVID-19 PCR positivity. This provided a valuable opportunity to study affected children, including asymptomatic and mildly symptomatic ones, who were typically not hospitalized in other countries.

### Characteristics of Pediatric Patients with COVID-19

Among our patients, using a strict definition of “asymptomatic”—defined as lack of clinical symptoms, radiographic findings and laboratory abnormalities—43 (38.7%) children were truly asymptomatic; an additional 19 children showed no symptoms but at had least one abnormal inflammatory marker, reflecting a systemic proinflammatory state. Hence, when only clinical symptoms were used to categorize our patients, 62 (55.8%) were asymptomatic compared to the 14.9% to 28% reported in the current pediatric COVID-19 literature [11-14].

Our study cohort spanned 22 nationalities. This mirrored the UAE’s diverse population, encompassing an expatriate population of 88% [15]. The vast majority of our patients acquired COVID-19 infection from close family contacts. In our study, we reported 93.7% family clustering, which was higher than the 75% to 90% rate previously reported among children [11,12,16,17]. We theorize this may be due to the strict quarantine measures imposed by local authorities and pre-emptive closure of schools and nurseries at the start of the outbreak, hence limiting wider community transmission. Pre-existing medical conditions were reported in up to 25% of children with COVID-19 in a European multicenter study. Most of our patients were previously healthy, and only 3.6% had a history of asthma; this was lower than expected, given the prevalence of asthma in the UAE was reported at 13% [18,19]. It was thought that asthma predisposes children to increased susceptibility and severity for COVID-19 infection. A few other studies similarly reported low asthma comorbidities among patients with COVID-19 infection [20,21]. Early results from the literature suggest that one of the inhaled corticosteroids (ciclesonide) exhibited antiviral efficacy and inhibited SARS-CoV-2 replication [22,23].

Fever and cough remained the most common presenting symptoms for COVID-19 infection among children in various studies, including ours. Published pediatric studies reported fever in 47% to 59% of patients and cough in 37% to 55% [12,14]. This rate was much higher than our observation, reflecting the high number of asymptomatic and mildly symptomatic children in our study. None of our patients had dyspnea or tachypnea at any point of their stay. Anosmia had been reported more frequently in adults than in children, and it was more prevalent in our study (4.5%) than previously reported (1%) in children [24]. Gastrointestinal symptoms, including vomiting, diarrhea, and abdominal pain, were infrequently presented both in our study and in other pediatric COVID-19 studies [12,13]. None of our patients presented with symptoms of multisystem inflammatory syndrome, although 1 child had a nonspecific rash.

Fewer children with COVID-19 had laboratory abnormalities compared to adults. A meta-analysis of pediatric patients with COVID-19 reported leukopenia or lymphopenia in 28.9% and increased creatine kinase levels in 20.1% as the most common laboratory abnormality [13]. Elevated LDH levels were the most common laboratory abnormality reported in our study, and it was more frequent than that reported in a meta-analysis by Ding et al (42.5% vs 8.3%) [13]. Increased LDH levels have been associated with severe COVID-19 infection [25]. Consistent with this finding, we reported higher LDH in symptomatic children.

Although most studies of COVID-19 infection report lymphopenia and neutrophilia, none of our patients had lymphopenia; however, 12.4% were neutropenic at presentation. It was likely that lymphopenia was a marker of severity of COVID-19 infection; however, since none of our patients had severe disease, coupled with immature immune systems in children, further studies are needed.

Chest CT scans were frequently used during the early phase of the pandemic. A systematic review of imaging findings in children with COVID-19 reported that up to 60% of asymptomatic children had abnormal CT scan findings, including ground-glass opacification and consolidation. However, only 2 children who had progressive symptoms underwent chest CT scans in our study to reduce unnecessary radiation exposure. Follow-up studies often demonstrate resolution of earlier abnormal chest imaging findings, suggesting that long-term pulmonary damage was unlikely [26,27].

Several adult and pediatric studies have shown high rates of concurrent antibiotic use in managing COVID-19 infection [12]. Antibiotic use for bacterial coinfection in our study was extremely low since most of our patients were clinically well.

### Duration of Viral Shedding in Asymptomatic and Symptomatic Patients

Very few studies have evaluated the duration of viral shedding in patients with COVID-19 infection. One study in asymptomatic adults reported a median duration of nasal COVID-19 shedding of 19 days (range: 15-26 days), with asymptomatic group patients shedding for a significantly longer duration than those with symptoms [28]. Studies in children suggested a mean duration of viral shedding of 10 days, with prolonged shedding occurring in children with moderate symptoms compared to those with mild symptoms [11]. Among our study population, viral shedding continued for a median of 10 days (range: 1-39 days), without any significant difference between symptomatic and asymptomatic children.

Several challenges have emerged during the COVID-19 pandemic for children and youth including heightened anxiety, disrupted routines, academic and social stresses associated with school closure, and increased risk of domestic violence and abuse [29]. Hospital admission of our studied subjects despite a lack of clinical need for most of them (as per the national COVID-19 guidelines at that time), would most likely have mounted the level of already existing COVID-19 pandemic stress regarding health and well-being, in addition to developing separation anxiety (school-age children isolated from parents), reduced access to psychosocial support, and boredom.

Digital approaches including telemedicine are rapidly established during the current COVID-19 pandemic. They played a major role as a reliable resource to overcome restrictions and challenges, and increased access to effective, accessible, and consumer-friendly care to more patients and families [30].

Currently, children with confirmed or suspected COVID-19 can be isolated at home, assessed, and managed by telemedicine consultation rather unless there is a clinical need for face-to-face consultation or hospital admission.

### Study Limitations

Our study's primary limitations were related to the relatively small study population and to the limitations inherent to a retrospective chart review. The changing treatment guidelines by local recommendations precluded any evaluation of treatment efficacy among our patients who received treatment.

### Conclusions

Based on our analysis of pediatric patients with COVID-19 from a highly diverse population in the Middle East, we found that many of our demographic and epidemiological findings were similar to those previously reported for COVID-19 infection in children worldwide. However, we observed a higher frequency of asymptomatic and mildly symptomatic children with COVID-19 and some differences in laboratory abnormalities compared to other pediatric studies. Our findings of a similar duration of viral shedding in symptomatic and asymptomatic children highlight the possibility of virus transmission by asymptomatic children, hence reinforcing the importance of continued social distancing, universal mask use and comprehensive contact tracing to control COVID-19 outbreaks once children return to schools. 

## Abbreviations

ALP: alkaline phosphatase
ARDS: acute respiratory distress syndrome
AST: aspartate transaminase
CDC: Center of Disease Control and Prevention
CRP: C-reactive protein
CT: computed tomography
CXR: chest x-ray
FiO_2_: fraction of inspired oxygen
LDH: lactate dehydrogenase
ORF: open reading frame
PCR: polymerase chain reaction
RT-PCR: real-time reverse-transcriptase polymerase chain reaction
SARS-COV-2: severe acute respiratory syndrome coronavirus-2
SpO_2_: oxygen saturation
UAE: United Arab Emirates
